# Mr. Chevalier's Cases of Dissection

**Published:** 1802-05

**Authors:** T. Chevalier

**Affiliations:** South Audley Street


### To Dr. BRADLEY.

Dear Sir,

I Have within the laſt month been called upon to open five perſons, who have died of a diſeaſe which had been but little ſuſpected, and which appears to be a variety of the *Catarrhus Suffocativus*. As theſe caſes occurred ſo nearly together, I fear the complaint may be epidemic, and therefore feel it my duty to communicate to practitioners the reſult of the Diſſections through the medium of your uſeful Journal.

Two of theſe caſes were in adults, and three in young children. The firſt was that of a young man, about twenty years of age, who was under the care of my friend, Mr. Reid, of Chelſea. The diſeaſe at the beginning, Mr. Reid informs me, had every appearance of an attack of mild Typhus. On the two firſt days there was no affection of the cheſt whatever; on the third day the patient complained of pain in his ſide, to which a bliſter was immediately applied. The ſymptoms of general debility were ſuch as to forbid the uſe of the lancet. On the morning of the fourth day he ſeemed relieved, but in the evening great difficulty of breathing came on; he was, however, able to walk out of his room as late as nine o'clock. After this he went to bed, but in the middle of the night he awoke, complaining of a ſenſe of ſuffocation, became exceedingly irritable and paſſionate, and died at three o'clock in the morning.

We examined the body about ſeven hours after death, and on opening the cheſt, it was obſerved that the lungs did not collapſe; we therefore expected to find them adhering to the pleura, but this was in no degree the caſe. The lungs were entirely free from any mark of inflammation, or alteration of ſtructure; nor was any fluid effuſed into either ſide of the thorax, or into the cavity of the pericardium. On laying bare the trachea, and making an inciſion into it, an immenſe quantity of thin mucous guſhed out, with which both it and the bronchiæ

Ddd2 were were completely filled; a confiderable quantity was alfo preffed from the air-cells of the lungs. The inner membrane of the trachea and its branches appeared much more vafcular than ufual; there was no effufion of coagulable lymph upon it, but a few flocculi of that fubftance were obferved in a part of the mucus which was contained in the fubdivifions of the bronchiæ. The head was opened, and the dura mater was found at one part fomewhat inflamed. The abdominal vifcera were all in a healthy ftate.

The fecond patient was between thirty and forty, and was likewife feen by Mr. Reid, but not till about ten days after the commencement of his indifpofition. At that time he appeared extremely languid, had a fmall weak pulfe, fome difficulty of breathing, and rigors. A large blifter was applied to his cheft, and an emetic, and other fuitable medicines exhibited. He went on for a week without any material alteration in the fymptoms. On the eighth day of Mr. R's attendance, his breathing became more oppreffed, and he complained more of cough, and pain over the whole of his cheft. As his debility did not appear greater than it had been, fix ounces of blood were taken away. In the evening he became more reftlefs, and fomewhat delirious. About five the next morning he awoke in a violent fit of delirium, and died, app rently fuffocated, in about half an hour.

The lungs in this cafe did not collapfe when expofed, but they were not in the leaft inflamed, or difeafed in their ftructure. The trachea and its branches were completely filled with the fame kind of mucus as in the former cafe; fome was found in the air-cells. The membrane lining the trachea had alfo the fame appearance of increafed vafcularity. The head was opened, but no mark of difeafe was difcovered in it, or within the cavity of the abdomen.

The two next cafes were in children about four or five years of age. I was informed of both that they had flight pectoral affections for a week or ten days, which had excited no particular alarm, but were followed at length with a degree of ftupor, which gave rife to a fufpicion that mifchief had arifen within the head; and they were both examined after death at the requeft both of the parents and the medical gentlemen who had vifited them, under that impreffion. Both of them died convulfed. There was no difeafe whatever within the head of either; but in both, the trachea and its branches were filled with thin mucus, and its inner membrane was flightly inflamed. The lungs collapfed but very inconfiderably when the thorax was laid open, and yet very little mucus was contained in the air cells.

The

The fifth and laft cafe I had an opportunity of feeing before death, as the patient was the child of a relation of my own. It was about two years old, and had been attacked, rather feverely, with the meafles, for which it had been very properly treated by the apothecary of the family. The fourth day after the eruption I called at the houfe, and found the mother diftreffed with a fufpicion that the child was labouring under water in the head, as it had become ftupid, was laying with its eyes half open, and took no notice of any thing. But on examining the eye, the pupil contracted properly, and the child, when roufed, appeared perfectly fenfible. The next day thefe fymptoms continued; its countenance was bloated but florid; the breathing was fhort, but not with that fhortnefs which arifes from inflammation, and is produced by a fenfe of pain on expanding the cheft, but which feemed as though the cheft did not contract properly in expiration. It coughed with a rattling noife, but not frequently or violently: Its pulfe was very frequent, and feeble, but not at all hard. Having fo recently examined the former cafes, I fufpected this to be like them, and recommended the exhibition of oxymel fcillæ, with vinum antimonii, to excite fome naufea, and that in the intermediate times light nourifhment and white wine whey fhould be given. Thefe remedies were employed, and a blifter applied over the fternum. The medicine did not produce vomiting at firft, but appeared to relieve the breathing for a time; the next day, however, it became more laborious, and the medicine was given in larger dofes, and more frequently, fo as to produce repeated vomitings, by which a very great quantity of mucus was thrown up, and the breath was the following day fo much relieved, that we thought there was every reafon to hope the child would recover. It fat up, took notice of its play-things, and took nourifhment willingly. But on the eighth day it became extremely languid, and died, after a fhort fit, apparently exhaufted, on the morning of the ninth.

On opening the cheft the lungs did not collapfe, but were free from every appearance of inflammation or adhefion. The bronchiæ were filled with mucus, and the trachea undoubtedly had been fo; for its inner membrane was exceedingly red, much more fo than in either of the former cafes; and the mucus in the bronchiæ had the fame appearance as in them, with flocculi of coagulable lymph intermixed in it. In this cafe I conclude the mucus was for the moft part difcharged by vomiting in confequence of the medicines that were given; but the air-cells had not recovered their elafticity, which accounts for fome fhortnefs of breathing continuing afterward. The child, however, would in all probability have recovered, had had this been the only diseafe; but the measles having preceded it, the strength was borne down, the powers of Nature exhausted, and the feeble frame was unable to resume sufficient vigour to repair the injury it had received.

Whether the disease I have now descr[illegible]bed is to be considered as the true *Catarrhus Suffocativus*, I leave you to decide. It might perhaps, without much impropriety, be considered as a *Coryza Trachealis*; for it was not attended in either of the cases with those general inflammatory symptoms which usually attend Catarrh; but seems to have consisted in only as much inflammatory action on the inner surface of the trachea as would augment its secretion of mucus, (just as a Coryza does that of Schneider's membrane) without producing any considerable degree of febrile action in the system. The principal constitutional symptoms came on late, and appear to have been rather symptoms of irritation and debility, than of inflammation, and were probably in a great measure the effect of the disease, and of the imperfect degree in which the blood received oxygen from the air. As long, however, as the absorbents took up the mucus, and the air could get a passage into the lungs, their functions went on; and as there was not inflammation enough to produce much pain, the symptoms excited but little alarm. But when the secretion of mucus augmented so as to exceed the action of the absorbents, the trachea became filled, and life was presently extinguished.

I shall be happy if my having troubled you with this Paper should be the means of bringing forward any fa[illegible]ther information on the subject, or of calling the attention of practitioners to the attack of an insidious but very formidable disease.

I remain, &c.
T. CHEVALIER.

*South Audley Street.*

